# Finding Ramón: Navigating Failure and Luck in Mexico

**DOI:** 10.1007/s10612-025-09851-2

**Published:** 2025-12-01

**Authors:** Gareth A. Jones

**Affiliations:** Department of Geography & Environment, The London School of Economics & Political Science, Houghton Street, London, WC2A 2AE UK

## Abstract

Failure is taboo. Yet as researchers we often encounter failure in the field and engage in confessional conversations with colleagues about ‘what went wrong’. Failure is regarded as an unfortunate and unproductive part of research. Better luck next time. In this paper I offer an autoethnographic narrative, tracing the attempt to find Ramón to conduct a longitudinal life history. A 20-something member of a group of street youth increasingly involved in gang and criminal activity when last we met, efforts to reconnect with Ramón more than a decade later were unsuccessful. I reflect on this apparent failure and how it provoked an unexpected appreciation of luck in ethnographic research.

“Ever tried. Ever failed. No matter.

Try again. Fail again. Fail better.”

-Samuel Beckett (1983: 1)

## Introduction

The ERC-funded GANGS project^1^ has, as one of its aims, to compare the life paths of gang members across the world, seeking to understand how individuals are motivated to join, remain part of, or perhaps leave gangs. The project adopted longitudinal life history as part of an ethnographic approach to tease out how interlocutors make sense of their gang involvement, past, present, or future. Invited to return to Mexico, my primary research site, to conduct a repeat life history, I opted to ‘find Ramón’. A charismatic 20-year-old when first we met, Ramón had been a key interlocutor for a project on street identities that I carried out in the city of Puebla between 2006 and 2009. He had claimed then to have been a member of multiple gangs in Puebla and Mexico City, and had certainly become more involved in street crime during my research, and was arrested shortly after our last meeting, and, it was rumoured, sentenced to 20 years in prison.

The opportunity to reconnect with Ramón was exciting for several reasons. First, I was intrigued to find out how Ramón had fared in the intervening years, whether the friendly charm of our earlier encounters had given way to a steelier, tougher outlook, and how far any changes might be related to prison or gang involvement. My expectation was that Ramón had continued the lifepath that I witnessed when last we met. He had plenty of experience with low-level street crime, but few of the skills required to be drawn into a major criminal organisation, unless he had picked these up in prison. More likely, I thought, I would find Ramón hustling odd jobs, still using drugs, intermittently moving between crime and prison, and lacking any stable relationships. Second, I was curious how Ramón, by now in his late 30s, would reflect on his earlier life and how far this would tally with the self-image presented in the life history interview conducted in 2006. I had long wondered about the veracity of Ramón’s original narrative and to what extent he had exaggerated or even made up some of his accounts of involvement with gangs. And while I did not expect the new life history to completely contradict the original narrative, I thought Ramón might retell events differently or miss some elements entirely (see Rodgers, 2021; Roks, 2021). Rather than an attempt to deceive, I had interpreted some of the more spectacular elements of his life history to be an effort to appear more streetwise than others, underpinned by a need for stimulation—via drugs, crime, violence—and the psychological toll of loneliness and boredom (Jones et al., 2007).

Much as it was my intention to consider these themes in the follow-up life history, this is not what happened. Finding Ramón proved far more challenging than I had anticipated. Looking back, it is difficult to imagine why I had thought it might be otherwise. After all, numerous researchers on gangs have described the difficulties of entering the field, the time it takes to build trust and the sense that everything could all fall apart with one misstep or change of circumstances (see, for example, Rios, 2011; Roks, 2021). Yet, as Contreras has argued in developing the notion of what he calls “broken ethnography”, researchers rarely share the intensity of feeling provoked by impending or actual failure or the risks of perseverance. Rather, he writes, “in the academy, everyone loves an ethnographic hero. An ethnographer who struggles with a broken ethnography is seen as a failure, as weak” (Contreras 2019: 162). Better, then, to emphasise the breakthrough when the researcher “got lucky” and it finally ‘came together’, as Contreras concedes it did eventually for him. In so doing, and perhaps especially for “risky” ethnographies (Koonings et al., 2020), the trope of the plucky hero defying the odds, with its masculinist and occasionally racialised stereotypes, is sustained (Rios, 2011). But what if, as in the case of my attempt to find Ramón, there is no ‘hero’ moment? This paper provides an autoethnographic narrative of failure. Specifically, it is about the twists and turns of trying to reconnect with Ramón, and in so doing how I was obliged to reflect on the fragility of social relations, the difficulties of carrying out longitudinal studies, and how ethnographic research often relies on luck more than we are typically comfortable to admit to ourselves or others.

## Leaving the Field, Losing Ramón

It is important that I begin by explaining how I left the field, and the last time that I saw Ramón. This was in 2009, and I had spent nearly two years hanging around Las Jardineras, an area close to the Mercado Miguel Hidalgo in Puebla, which was occupied by a group of about 35 street-living people at the centre of which was Doña Chita and her sons, Alvaro, Alfonso, Nacho, and Lucio.^2^ Most days were spent attempting to gain money, buy food, stay clean, play games, exchange gossip, and acquire and then sleep off the effects of drugs, mostly PVC solvent. The relaxed atmosphere at Las Jardineras was occasionally punctuated by moments of drama and agitation, for example when police or state security agencies were active in the area. These actions often focused on the market which was controlled by a social movement known as Unión Popular Vendedores Ambulantes (UPVA)-28 de Octubre, which had an antagonistic relation with the state.

Mexico’s ‘war on drugs’ was in full swing at the time. The Hidalgo market area had a reputation for the presence of criminal organisations linked with contraband, kidnapping and human trafficking, and street robberies. Nevertheless, the visible presence of the police was rare except during the sporadic raids that sometimes also involved federal agencies. And levels of violence in Puebla were generally lower compared with other Mexican cities, despite a nation-wide rise in the number of homicides from around 10,000 in 2006 to over 26,000 in 2011. This relative calm was largely explained by the ‘public secret’ of a pact between cartels and senior politicians that made the city a safe(r) space for cartel members’ families in return for keeping public violence to a minimum. This agreement would break down in 2010 with a change of government and the arrest of leading cartel figures, resulting in an uptick in homicide, disappearances and political assassination (Zavala, 2023). Over the intervening decade, the city would experience changes to the control and form of the drug market—notably the increased availability of methamphetamine and crystal meth—as well as the growing involvement of organised crime in oil pipeline thefts (*huachicoleo*), and a greater presence of gangs.^3^ Increases in homicides in the state, and especially the metropolitan region of Puebla, would more than double compared with a fifteen percent increase nationally, resulting in just under 37,000 deaths per annum from 2019 to 2022. Since my original fieldwork and in line with many other states, public security in Puebla has become militarised with the involvement of the army and the National Guard, although neither were present near the Hidalgo market when I returned in July 2023.

When I would sit with the Jardineras group in 2009, however, these shifts in the governance of illicit markets or security measures seemed of only passing significance, even to Ramón. An almost constant presence at the beginning of my first stay in Puebla, over time Ramón had seemed increasingly frustrated by the mundaneness of life around the Jardineras and would disappear for days at a time. Our conversations also changed. At the beginning, our chats had been short, revolving around topics such as football, clothes, my family (never his), but slowly the topics began to stretch to more serious subjects and were expressed more discreetly. Sitting away from the others, Ramón would ask my views on drugs, girls and familiarity with guns, sharing the information of where he could get one and for how much. He was clearly not well informed about weapons and confessed to having shot himself in the leg.^4^ On one occasion, I showed him a large scar on my right calf, joking that I too had been shot. While I expected Ramón to laugh—with no evidence of an exit wound my scar was clearly not caused by a bullet—he took the story seriously and regarded it as something that drew us closer together.

Ramón also seemed increasingly less engaged with the Jardineras group and spent more time with El Chino, a member of a local gang, who had served prison time for armed robbery and burglary. Late one morning, towards the end of my stay in Puebla, I was sitting with Ramón and others at the Jardineras when I noticed that he was constantly looking around. Suddenly, he sprang up and ran across the road towards the market, before returning briefly, only to then jump on the back of a motorbike that quickly sped off. It was the first time that I had seen this happen and did not know who the bike belonged to. A few hours later Ramón returned, jumping off the same bike and looking very pleased with himself. Yet, while ordinarily anything that punctuated the boredom of hanging about the Jardineras would generate animated discussion, Ramón offered no explanation of what had happened, and nobody seemed to ask.

A few weeks passed. I went to the Jardineras in the early evening. It was dark and it had been raining which added an unpleasant smell from the trash and excrement. As I scanned who was about, I noticed some strangers standing off to one side. As I wanted to catch up with everyone on the events of the day, I did not think any more about them. After chatting with people for 30 minutes or so, I noticed that some of the Jardineras group had retreated to the back of the gardens. Usually when this happened it was to arrange the sharing of money or drugs. But a short time later, a motorbike drew up to the curb and one of the strangers approached the rider who dismounted and the two looked about. Then, from the back of the Jardineras, Lucio approached and we started to chat. A lot shorter than me, I had to lean in as he whispered “Garet, those guys are bad, go now. Leave”. Lucio was always friendly, but this time his tone was frightened. He reiterated, “something is going to happen. Go”.

It was the last time that I would see Ramón and many of the others as a short time later I left Puebla and returned to the UK. In the months that followed I reflected on this moment–Leaving the field so abruptly in some sense provided a definitive close, but it also left a lot of issues hanging. Was Lucio’s fear justified? Was Ramón caught up in something more serious than “*cotorreo*” (pulling pranks) and opportunistic street crime? A few weeks on, I received a newspaper clipping from Elsa who had been a research assistant with the project in Puebla. It showed a photo of Ramón who had been detained by a municipal police unit, the *Grupo Táctico Guardian*, for being under the influence of PVC solvent and possession of a long-blade knife. The penalty for possession of a dangerous weapon was a few months detention at most. It all seemed like business as usual for Ramón. So, I was not surprised when a few years later Elsa again sent news that she had bumped into El Chino who had told her that Ramón had been arrested (email 10/7/2012). Except this time the sentence, El Chino had claimed, was 20 years in prison. Elsa and I speculated that this length of sentence suggested “something very serious”, possibly either homicide or involvement with organised crime (email 19/7/2012). Over subsequent weeks, Elsa returned to the Jardineras to look for Doña Chita who might be able to corroborate El Chino’s account, but nobody was around. Combined with the circumstances of my departure, our sense was that the Jardineras was no longer conducive to the extended hanging out that had marked the original research.

## Who is, or was, Ramón?

Ramón was one of the first people I met at the Jardineras. As with many of the others he was cautious but friendly. Although short, perhaps around 1.65 m, Ramón was strong and would often display his muscles by wearing cap t-shirts and vests. He was also fit. In one of the early meets some of us decided to play football on some waste ground. The kickabout was chaotic, punctuated with petty squabbles. Ramón was competitive, uniquely skilful and kept playing when everyone else had lost interest. Throughout the two years that I interacted with Ramón, this demeanour and his physical appearance changed little despite the consumption of drugs and uncertain diet (Herrera et al., 2009).

According to the life history collected in 2006, Ramón was born in Tierra Blanca in the state of Veracruz. He claimed to have family there still, although it was never clear what had happened to his parents nor why he had come to Puebla where he had nephews and therefore, somewhere, a sibling or siblings. Rather, Ramón gave the impression of getting by on his own. From age 10 he had worked, selling compact discs at the *Fayuca* (contraband market), as a causal labourer, and at an amusement arcade. The most amazing claim is that aged around 13 he had spent time as a diver at the famous La Quebrada cliff in Acapulco. His explanation for why he had left some of these jobs, including diving, and why he preferred washing windscreens or being a *chalán* (fare collector) on the microbuses, was “I get bored”. Indeed, as evidenced by his life history and observation, the twenty-year old Ramón was in a perpetual search to avoid being bored. Girls also featured prominently at key moments in his life history—meeting a girl in Querétaro before being run out, ending up in Mexico City, meeting a girl, getting to Monterrey because of a girl…

Relations with girls appear also in Ramón’s account of his involvement with gangs. Aged 15 or 16 he claimed to have been part of a gang called “Los Osos Leyes” in the *barrio* (neighbourhood) of Tepito in Mexico City, introduced through a girlfriend. He described the gang as one of the toughest “*de antes*” (from before). According to his life history, Ramón was obliged to fight for the gang for self-protection and to defend territory, adding that “we would fuck them up, whatever, beatings, stabbing, belt straps, bottles, you know, whatever happens”. At this time, Ramón claimed to have carried a 0.38 revolver although he made no mention of having used it and said that he had sold it in a moment of religious repentance.Nevertheless, moving to Puebla, Ramón quickly formed a gang in the *colonias* (residential neighbourhoods) close to the Wholesale Market. This gang was “less heavy” than in Tepito, and had around 22 members, including a few in prison, and seems to have been mostly social, dedicated to “*putazos*” (messing about) and “*mazapanzos*” (dishing out beatings).

But there was some evidence that circumstances were changing. In 2008 I took a photo of Ramón. He was wearing a NY-logo baseball cap and dark sunglasses. He insisted on being bare chested. It was obvious why. A large tattoo of the Santa Muerte, a skeleton figure with a scythe and outstretched wings, adorned his chest, and a Santa Muerte medallion hung around his neck.^5^ By no means exclusive to gang members, the size of the tattoo was dramatic. Moreover, while previously we had talked about the numerous self-made tattoos such as a “VL”—for *Vatos Locos* or “Crazy Ones”—on his wrist, a photo I had taken of him in 2006 showed no tattoos on his chest, and he had never mentioned this larger, more professional one before. Neither had he mentioned anything in 2006 about connections with *Sureño* gangs in Los Angeles, something that he talked about in 2008, claiming that he had been in LA and spent time in prison there for having gang links.^6^ I was unsure at the time if this ‘reveal’ was an invention or a sign that Ramón was becoming more confident in confiding his involvement with crime. There was also the suggestion that he was acquainted with HEM26, possibly introduced through El Chino, a gang that was extending its presence across an arc of *colonias* to the north of Puebla, from San Felipe Hueyotlipan to San Pablo Xochimehuacán, the latter including the Wholesale market.

## Searching for Ramón

The GANGS project’s invitation to conduct a new life history presented the chance to reconnect with the Jardineras group and address some of the questions hanging over Ramón’s relations with gangs and criminal organisations. I had often wondered how much of the account in the 2006 life history interview, as well as other conversations, had been imaginary or embellished. It seemed highly unlikely that he had spent time in Acapulco as a diver. Would he mention joining a *Sureño* gang or recall turf battles in Tepito? And how might he explain his multiple arrests and experience of prison?

An immediate issue, however, was that I had not been back to the Jardineras since 2009, having been caught up with other life events – the birth of a child, my partner’s cancer diagnosis, the death of parents, new career roles and other research projects. I had remained in touch with Elsa who had joined a non-governmental organisation, *Junto con los Niños* (JUCONI), working with child rights before leaving to work at the state university. An internationally respected source of practice for its work with street-involved children, for over 20 years JUCONI was directed by Alison Lane. I had got to know Alison well and I had drawn many times on her connections with human rights organisations and the criminal justice system. During my periods away from Puebla, Alison remained an invaluable source of information and advice. In June 2015, however, she was bitten by a Brown Recluse spider, went into septic shock and died a few weeks later. I was devastated at losing a friend and, more coldly, a link back to the field.

Alison’s death was emotionally difficult but not the only challenge that had to be addressed if I was to find Ramón. In 2009 none of the Jardineras group had mobile phones and many were functionally illiterate. To find Ramón, then, would it be a case of just turning up? Would hanging around the Jardineras be feasible given the circumstances of my departure? As I started to think how I might go back, more bad news arrived. In casual conversation a friend mentioned that the Jardineras area had been remodelled since I had last been in Puebla. An online search confirmed that the Jardineras had been cut in half by an urban light rail line and terminus station. Inaugurated in 2017 but with presumably too few passengers wanting to travel between the archaeological zone of Cholula and the sprawling Hidalgo market, the line closed in 2021. If my original research in Puebla had been a ‘street corner’ study of sorts, there was now no longer a street corner but an abandoned station. My only hope was that one or several of my previous interlocutors might still be about, or that the ever-watchful UPVA-28 de Octubre might know of their whereabouts.

As a first step I messaged Elsa, who suggested that we try to re-connect with Nacho, Doña Chita’s youngest child, who had been a ‘star’ of JUCONI’s residential facility for at-risk youth. In October 2022, however, Elsa reported back that JUCONI had lost contact with Nacho. Nobody had seen him for at least three years. Eventually, I would discover that he had become a heavy drug user, spent periods in “*anexos*” (rehabilitation centres), lived on the streets, become involved with crime, and was now in prison. It was to become a familiar pattern as I learned more about what had happened to the Jardineras group.

Another attempt to locate Ramón was through a colleague in Mexico City, who told me that a former student was now a senior politician in Puebla. With the ability to expedite access to state records, we asked this contact to check if Ramón might still be in prison. The response came a few days later and was definitive. No record existed of Ramón in any state facility. A follow-up search with the State Public Prosecutor’s Office also proved fruitless. I pondered the accuracy of the records, wondering whether Ramón might have been incarcerated without formal sentence or under an incorrect name. To build trust, the original research had relied on nicknames or first names for identification. Unusually, I had a surname for Ramón, but I also knew that surviving on the streets sometimes meant obscuring one’s identity from social, religious and NGO workers, the police, and probably researchers. Had Ramón given a false surname to me or the Public Prosecutors Office? The surname I had was different from the name that had appeared in the newspaper clipping of his earlier arrest. Or had the rumoured 20-year sentence been made up or exaggerated, and Ramón had served a shorter tariff and been released?

Adding to the mystery, my colleague in Mexico City contacted a friend who worked for the federal government and who offered to conduct a search for Ramón. This produced 6 possible Ramóns, each with a location. But it seemed only one was in Puebla; in a prison in the municipality of Huehuetlán about two hours south of the city. Yet this information was confusing. While the text identified this Ramón’s location as Huehuetlán a cross check of the geographic identifier code indicated the municipality of Tepeyahualco, near the border with Veracruz, and not far from Tierra Blanca, Ramón’s birthplace. Even more tantalising, with one digit changed the code would indicate Tepexi de Rodriguez, which has a large state prison. But the data put this Ramón’s age as 59! The only feasible candidate from the 6 was aged 35 but located in Chihuahua.

An alternative was to approach directly the state carceral system, the *Centro de Reinserción Social San Miguel*, known as the CERESO. If Ramón was still imprisoned, this is most likely to be where I would find him. Entering the CERESO, however, would be tricky. Formally, access is only permitted to family or people nominated by the prisoner or their legal representatives. And I recalled Alison telling me that access had become increasingly difficult even for organizations such as JUCONI. The Mexican state was wary of human rights groups and journalists, especially given conditions at a facility designed for two thousand inmates that now held closer to five thousand, and which was the subject of repeated scandals concerning governance and violence. Even assuming Ramón was in the CERESO, access would rely on resolving a Catch 22: to be admitted Ramón would have to nominate me as one of his five permitted visitors but he would be unaware that I was looking for him and I had no way to alert him. Even then, there was the possibility that he might not want to see me, not an unreasonable prospect given the length of time since last we met, and if he had become involved with organised crime.

## July 2023: First Revisit

I returned to Puebla in July 2023. An initial visit to Mercado Hidalgo suggested a bad omen in the search for Ramón. There was nobody hanging about the remaining Jardineras space. Nor did I recognize any of the windscreen washers or street traders. Most seemed too young to have been around in 2009, and those I spoke with did not remember Ramón or seemed to be guessing as to his identity. There was greater success with peoples’ recollection of the Jardineras group and it was possible to piece together a rough list of what had happened to some of them. It was a depressing summary. Shortly after I had departed, Estela, the partner of Doña Chita’s son Alfonso, died from drug use, not long after having abandoned her daughter, who had been looked after by Doña Chita. Alfonso had disappeared and was presumed dead. Doña Chita herself and another of her sons, Alvaro, had gone away, the destination variously said to be Guerrero, Jalisco or Veracruz. Alvaro had split with Amparo, and her whereabouts and that of their son, Juan Alvaro, was unknown. El Chino had got in to trouble with the traders for being drunk, using drugs, and being regularly prone to outbursts of violence. One source thought he was dead. El Huevo was hit by a van at the junction and killed.

I was given two pieces of more positive advice. First, to leave my mobile number with a market trader, El Señor de los Discos. In his seventies, partially deaf, and just about making a living from selling pirate DVDs, he was said to be trusted by surviving members of the Jardineras group when they did swing by. Second, I was told that Lucio and El Pachón might be hanging out on two avenues near the city centre, 11 Norte/Sur and Avenida Diagonal. The prospect of contacting Lucio was attractive given he was the last person I had seen at Las Jardineras. And El Pachón I remembered was close to Ramón and had been in prison for small-scale drug dealing.

Over the following days, with Elsa’s son, Gastón, I walked 11 Norte/Sur and Avenida Diagonal. Conversations with stall holders, windscreen cleaners, street sweepers, and a randomly met Nicaraguan gang member were all fascinating in their own way but largely unhelpful. Numerous times people commented that a person resembling El Pachón was here ‘recently’ or would be around “*en seguida*” (shortly), a colloquial nicety implying a timeline from hours to weeks to never. It was also notable how difficult it was to judge someone’s age, especially given tough living conditions. Descriptors such as body shape and hair type were highly subjective. Lucio, when I knew him, was thin, short, with wavy hair, and El Pachón, darker skinned, with a broad nose, and body weight that fluctuated with drug use. A suggestion that a guy about the right age and height as El Pachón was hanging out at a corner raised hopes only to be dashed when he said his name was Tomás. This pattern repeated itself many times.

After a few days chasing shadows, I felt that I knew every windscreen cleaner and trader at every junction over a wide area. More promising, I now had a small network of contacts who offered to keep an eye open for Lucio and El Pachón. Even so, it was unexpected when one morning I got a call from an unidentified number. It was Lucio. He was using a borrowed phone having picked up my number from El Señor de los Discos. We arranged to meet a couple of hours later at the market. Lucio arrived, a little late, carrying a polystyrene tray with chilaquiles and a soda. I hardly recognized him. Estimating that he would now be in his late 20s Lucio looked to be closer to 50, with deep lines around his blood-shot eyes, a missing front tooth, and sallow skin. He walked with a pronounced stoop and slight limp, in part from a heavy rucksack that I learned later held all his possessions. Always ‘humble’ as he put it, Lucio was embarrassed at being dirty and after a fist-bump greeting insisted on washing his hands before a full *abrazo* (hug). Almost as if nothing had changed over the intervening decade-plus, we sat down on a concrete bench and talked. Lucio was edgy, constantly casting looks about, and after 20 minutes suggested that we move to somewhere quieter, concerned by the presence of a man close by talking on his mobile phone. Although over the coming days we would meet several times in the market, Lucio explained that he did not trust the traders, nor the lookouts of UPVA-28 de Octubre. Everyone was a “*ratero*” (thief), he said.

At this first meeting Lucio was emotional. The apparent joy at seeing each other after so many years was overtaken by him breaking down in tears as he recalled the events of the past decade. Indeed, for the first 20 minutes it was near impossible to understand what he was saying, his voice failing. The upshot, however, is that immediately after I left, “different people” had appeared at the Jardineras, whom I learned later were a kidnap gang. Shortly after, most of the group had shifted consumption away from solvents and toward crack and crystal meth. Lucio had become addicted to “whatever was put in front of me”, going on binges for days at a time, and eventually becoming estranged from his partner and two children. Addiction resulted in crime and a one-year jail term in a CERESO prison in Cholula, an experience that affected him deeply, breaking down to whisper that if he ever went back there it would be to die. Claiming not to have taken drugs since leaving Cholula, nine months earlier, the effects of longer-term addiction were evident in his repeated ‘nodding’ and jerky movements.

Lucio added to what I now thought of as the ‘death list’. El Sombras and El Box had died from drug use. He also confirmed that his brother Alfonso had died. And he corrected the claim that El Huevo was run over, insisting that he had been murdered, although by whom and why remained unclear. Contrary to the rumour that Alvaro had moved away with Doña Chita, he had met an older woman who he described as “*cristalizada*” (addicted to meth) and both now lived with her children near to Mexico City. Formerly close, Lucio felt abandoned by his brother, especially given the disappearance of Alfonso, his own imprisonment and that of his oldest brother Abraham and mother Doña Chita. This last piece of news sent Lucio into spasms. Although the account changed slightly with each telling, the essence was that in early 2020, Estela’s daughter, Jazmín, had accused Doña Chita of abuse and Abraham of sexual assault. Both were arrested, with Doña Chita sent to the CERESO prison in San Miguel and Abraham to Cholula. Jazmín subsequently ran away, failed to submit further evidence to the courts and had since disappeared. Even so, both Abraham and Doña Chita remained in prison.

During these conversations Lucio repeated the comment, “I am totally alone”. He had neither seen his mother nor brothers in over a year, and his children only very occasionally. He spent his days wandering the streets and sleeping at a hostel infamous for drug dealing and prostitution, and as a transit point for *Mara Salvatrucha* gang members from Central America passing through Puebla. As we talked and I got to know more of Lucio’s life, focusing on the task of finding Ramón seemed crass, and I felt a responsibility to help him instead. It was Lucio after all who had warned me to leave the Jardineras, and it seemed altogether too cynical to treat the relationship only as a gateway towards trying to produce a life history with Ramón.

That said, Lucio did provide me with some updates about Ramón. He confirmed that shortly after I had left Puebla, Ramón had become involved in armed robbery before eventually getting caught while holding up a bar near the city centre. But instead of 20 years, it seems Ramón was sentenced to less than ten as around 2019 he was out and in a relationship with El Pachón's girlfriend. Yet, it was not long before Ramón was back in prison, allegedly for assaulting a girlfriend in response, according to Lucio, to her unrelenting demands on him because of her crystal meth addiction. Nevertheless, a search of social and news media platforms and an archive of the city’s leading newspaper failed to produce any mention of these arrests. In an intriguing comment, Lucio suggested that Ramón had actually been keen to get back to prison. When the subject came up again a few days later, he repeated this interpretation, suggesting that Ramón was always “looking for fun” as he got bored outside of prison. Over a decade on from my initial encounter with him, it seemed that Ramón’s actions might still be motivated by a fear of being bored.

In talking with Lucio, however, the main insight was that Ramón was not in the CERESO in San Miguel but “por la frontera”, which he pinned down as “Tepeji”. A quick search suggested that this might be Tepeji del Rio in the state of Hidalgo, which has a large prison. Having been arrested in Puebla but in prison in Hidalgo now teased the possibility that Ramón had committed a federal crime. I started to think how I might approach the prison. As luck would have it, a friend claimed to know a lawyer in Hidalgo who might be able to help. To jump forward, however, within a few days I realized that Lucio’s “Tepeji” was most likely Tepexi de Rodríguez—the pronunciation is the same—a town close to Huehuetlán mentioned in the federal government database. About four hours from the city, far enough to be ‘the border’ in Lucio’s mind, Tepexi has a large modern state prison.

Before attempting to discover if Ramón was indeed in Tepexi, I decided to visit Lucio’s mother, Doña Chita, in CERESO San Miguel. If I could speak with Doña Chita it might be possible to set up a visit for Lucio, something he had been unable to do having lost all of his identity papers in the split from his partner, and she might also have news of Ramón. And so began an unexpected engagement with government bureaucracy. This took two forms. First, with the CERESO. Arriving at San Miguel I met with the Social Work team who confirmed that Doña Chita was there, as was Nacho, her youngest son—and Lucio’s brother—and formerly the ‘star’ of the JUCONI rehabilitation programme. The social worker provided advice on the wording of my letter to request access to the prison. I returned the next day to submit the letter, pessimistic that anything would happen and annoyed that I was leaving Puebla in less than a week.

Second, I tried to get Lucio some identification papers. We arranged to meet at the market, but he did not show up. Disappointed but not surprised, I returned over the following days to look for him more in hope than expectation. That hope, however, was raised when on one visit with Gastón I glimpsed a figure who resembled Moises, a former acquaintance from the Jardineras. A heavy drug user with a history of psychiatric detention, if this was Moises then he might know Lucio’s whereabouts, even possibly Ramón’s. Walking quickly, we tried to catch up but lost sight of the person in the maze of alleyways. Annoyed at the wasted effort, this failure was more absurd as this person, like Moises, had one leg and walked with the aid of crutches. And yet still he had eluded us. Although I can smile at the comedy of it all now, at the time I felt despondent as another opportunity to track down Ramón seemed to pass through my grasp.

I returned to looking for El Pachón. A few people had suggested that a person using this nickname hung out at Plaza San Pedro, a large mall about 3 kms from Mercado Hidalgo. Chats with people in the area revealed that a “*tragafuego*” (fire eater) called El Pachón occasionally slept in a nearby abandoned building. A visit failed to find anyone. Then a call from a trader told me that Pachón had turned up. I rushed over to see someone, the right height and build, and possibly the right age, and who when approached responded to being called El Pachón. He was polite but dismissive, claiming not to know anyone called Ramón. Was this just caution to a ‘stranger’? I was not convinced it was Pachón even when told by the trader that this person had spent time in prison, was a heavy drug user, and used to hang around the Mercado Hidalgo. I was even less inclined to believe this was El Pachón when he mentioned having a son in his 20s who was a state police officer. Disappointed at having failed to find the right Pachón I contemplated my options.

There seemed to be little choice but to wait for Lucio to call. Not wanting to lose contact, I had bought him a “*semi nuevo*” (i.e. stolen) phone and a data package. And I had given my number to people whom Lucio had indicated were trustworthy such as La Manita, owner of a kitchen in the market where we had eaten days earlier. But why had he not called or returned my messages? And then, Lucio did call, from a different number. A penny dropped. He did not know how to use a mobile phone or understand data packages and could not send a text as he was unable to read or write. This raised the difficulty of getting hold of identification papers especially as the starting point was an online database, the *Clave Única de Registro de Población* (CURP), which required inputting surname, place and date of birth. Repeated attempts to enter Lucio’s data onto the platform failed; whatever he thought was his name or birthday drew a blank. A solution was to get a copy of his birth certificate, which required a visit to the State Registry in the city centre. Off we went. Attended to by an official, digital and manual checks were made using numerous name and date of birth combinations. An attempt was also made to identify Lucio through records for his children. Nothing. The official broke the news. In her view Doña Chita had not registered Lucio’s birth, and his partner had not registered the children with his surname. Lucio’s argument that he had previously possessed a birth certificate got a sceptical reaction, the official commenting privately that it was probably fake. Her advice was to go to the sub-registry office in the delegation where Doña Chita had lived or, if no record existed there either, to start a process to register Lucio’s legal status from scratch. To do so, Lucio would need proof of address. Informed that Lucio slept in hostels or on the street, the official rolled her eyes and wished us luck.

Undaunted, we travelled out of the city to visit the sub-registry in Ocotlán. And then, by luck, we bumped into Lucio’s sister-in-law. Teia is Abraham’s wife, a regular visitor to the CERESO Cholula where he is detained and to CERESO San Miguel to see Doña Chita. More important, Teia was a human archive of what had happened to whom and when. And, at home, she had a plastic bag full of family birth certificates, health records, wedding photos, and legal documents. Teia thought that Ramón was in Tepexi, adding that he had lived with her and Abraham for some time before his recent detention, and had struggled to adjust to life outside of prison. Again, the term “boredom” was used. She had lost touch with El Pachón but last she knew he was alive, had split with his partner who had older children including one in his 20s, a state police officer! Had I met Pachón at San Pedro after all? Although years of street living and prison would change anyone’s appearance, I like to think that I have reasonable facial recognition skills, but also that he might have recognized me, at least as some strange foreigner from back in the day.

After spending several hours with Teia it was too late to get to the sub-registry. And while the bulging plastic bag had contained documents for Abraham, Doña Chita, Alvaro, Alfonso, Jazmín, and others, none referred to Lucio. He would have to return another time to start the process to register his birth, and then get the CURP, and then his ID card, and formally request access to the prison. Deep down I knew this was all very unlikely. The chances of Lucio ever reconnecting with his mother seemed remote. Just as we were about to leave, Teia’s phone rang. It was Doña Chita calling from prison. For the first time in over three years Lucio had a chance to speak with his mother and I could re-introduce myself, mention that I was trying to help her son and look for Ramón. The call lasted only a few minutes. It was my last day of fieldwork.

## November 2023: Second Revisit

A few weeks after returning to the UK after my first revisit to Puebla, I received a WhatsApp message from the CERESO San Miguel following up on my request to visit Doña Chita. The CERESO wanted to conduct a phone interview to gather more information about me and the purpose of the visit. Eventually, permission was given with condition that I present proof of my identity and links with Doña Chita, and that she agreed to see me. Things were looking up and I arranged a return to Puebla in November 2023. On my first day I met with Teia. She had not heard from Lucio since July, and his mobile number was no longer receiving calls. I felt guilty but decided to focus on seeing Doña Chita and search for Lucio later. When I did return to the market nobody had seen him. We never did reconnect, and I fear the worst.

After some bureaucratic games, I was allowed into the CERESO San Miguel. The conversation with Doña Chita was wide ranging, from her numerous health conditions, the death of Alfonso, to the possibility of submitting an injunction against unlawful detention. The response when I mentioned Lucio was to shrug. As conversation shifted to Ramón it was clear that her knowledge was limited but two important pieces of information were forthcoming. First, Chita was bemused at my description of El Pachón. She thought I was describing another interlocutor, El Picoro. Later, I would check my notes and photos from earlier the original fieldwork and confirmed the mistake. El Pachón and Picoro shared the same name, but it was El Picoro who was closest to Ramón, not El Pachón. To underscore the absurdity of my efforts, Doña Chita indicated that last she knew El Pachón was living with El Picoro “near the Pepsi”. This was helpful up to a point. The ‘Pepsi’ is a vast area of colonias and factories, and without a specific contact of little to not use. And second, Doña Chita was convinced Ramón was still in Tepexi. Could he be in prison but not in any government database? With the help of Elsa, I met a social worker who had worked with the prison service. She confirmed that prisoners were dispersed to CERESO Tepexi during the COVID pandemic as an alternative to President Andrés Manuel López Obrador’s directive to release prisoners as means to ease COVID transmission and reduce the risk of riot. But Ramón would only have been included if he had been involved with serious crime and in any case the dispersal was temporary. A promise to check the database of her department provided no record.

On the hunch that Ramón might be in San Miguel, I tried one last throw of the dice and applied to visit him there. After a few weeks and back in the UK, an email from the Secretaría de Seguridad Pública informed me that my request was denied. It did indicate numerous names for Ramón on the prison records, including known aliases, but confirmed that he had been released in July 2020. There was no follow-on address. The trail had gone cold.

## Conclusion

For many and different reasons interlocutors disappear, so too do researchers, as do their contacts. This might be especially so when the research field is ‘risky’ and the lives of interlocutors uncertain. Yet, I had always subconsciously assumed that the Jardineras group that I had studied in 2006–2009 would reproduce itself in some form and that therefore I would be able to reconnect at some point. This proved to be wrong. Returning to the field after a decade-plus absence I quickly learned that nearly every one of my previous contacts had disappeared, died or were in prison. Far from reproducing itself, the Jardineras group had collapsed, principally as the result of a shift in drug use from solvents to crystal meth. Lucio was addicted to meth, so too Alvaro and his partner, and Teia’s sister, El Pachón’s girlfriend, and possibly Ramón. Drugs caused or contributed to the death of Estela, El Box, Sombras, and possibly Alfonso. Prison was not a new experience to members of the group; El Chino, Picoro, Pachón and Ramón had all served time before or during my original research. But I discovered that Doña Chita, Abraham, Nacho, Lucio, and Ramón, had been or continued to be in prison. Not only had I lost track of Ramón over the decade but (re)making connections with past interlocutors meant working out who had survived and how to reach them.

The response of many ethnographers to ‘losing the field’, or a research interlocutor, is to remain flexible, to rely on one’s experience and follow potential opportunities (Schoon, 2025). Yet, in practice this adaptability reminded me of Contreras’s desperate attempts to mend a “broken ethnography” in which he felt like “an ethnographic zombie with my arms out ready to rip into whatever came next” (Contreras 2019: 172). I frantically followed one lead after another, very nearly heading off to Tepeji del Rio in Hidalgo, searching across the city for El Pachón only to realise too late that I should have been asking for El Picoro, but then possibly meeting El Pachón without realising. It felt like luck was not on my side.

And yet, as Rivoal and Salazar (2013: 1) argue, luck is not accidental but requires “sufficient background knowledge, an inquisitive mind, creative thinking and good timing”. It takes a lot of hard work to be lucky. And I had (just) enough familiarity and contacts to follow circumstances in the ‘field’. Despite never sufficient to find Ramón, these resulted in a rich research experience. Reconnecting with Lucio was personally important but our conversations also drew out the fracturing of social relations around the Jardineras, the effects of crystal meth, and the trauma of repeated betrayals and social isolation. Aware from previous research how street youth valued official identity but took care on how and to whom names, ages, places of birth, were communicated, I now had first-hand experience of how readily someone such as Lucio could become invisible to the state. Trying to help a former interlocutor to acquire identity papers, meant a visit to Ocotlán that resulted in an impromptu meeting with his sister-in-law, and a phone call with Doña Chita, and the opportunity for my first visit to a prison in Mexico. The line between failure and good luck, or perhaps the randomness of luck and serendipity is extremely slight.

And if my luck did not yield the ‘heroic’ breakthrough I had hoped with regard to finding Ramón, then I can now piece together a reasonable timeline of his life since 2010. I can sketch his increased involvement with armed robbery and periods spent in prison. There is a suggestion that his offences might have been considered serious enough for him to be moved to another prison during the COVID pandemic, although remarks by others cast doubt on this progression from street crime. I have a sense too, from multiple conversations describing his propensity to gender-based violence, that the Ramón I knew back in 2009 had become an angry and less likeable person who has very possibly socialised to life in prison. And yet, he remains an enigma, an ever-fainter memory, who even with better luck I doubt that I will ever find.

## Data Availability

Due to the nature of the research and ethical considerations, supporting data for this article are not available.

## Footnotes

1 See the Introduction to this Special Issue and https://www.graduateinstitute.ch/research-centres/centre-conflict-development-peacebuilding/gangs-gangsters-and-ganglands-towards.

2 The names of interlocutors are pseudonyms, all nicknames are original.

3 Between 2018 and 2024, Puebla would rank eighth for political killings and see the emergence of 40 criminal groups linked with oil theft (ACLED, 2024). Cartel presence also shifted with the long-established Sinaloa and Beltrán Leyva organisations competing with new actors such as the Jalisco Nueva Generacíon and Los Rojos.

4 Ramón had numerous scars, apparently from a fight over a girl at a Los Tigres del Norte concert where he was set on by four guys who stabbed him in the leg, stomach and back, resulting in a month in hospital.

5 The Santa Muerte, the Saint of Death, is a syncretic folk Catholic figure usually represented as a skeleton shrouded in a cloak, to which adherents offer devotional prayers to gain protection, against bad luck or harm, find love or get revenge (Jones et al., 2007).

6 Intriguingly, Vatos Locos is closely associated with Los Angeles Hispanic gang identity.
